# Improvement of Disease Management and Cost Effectiveness in Chinese Patients with Ankylosing Spondylitis Using a Smart-Phone Management System: A Prospective Cohort Study

**DOI:** 10.1155/2019/2171475

**Published:** 2019-02-25

**Authors:** Xiaojian Ji, Yiwen Wang, Yingpei Ma, Zhengyuan Hu, Siliang Man, Ying Zhang, Kunpeng Li, Jinshui Yang, Jian Zhu, Jianglin Zhang, Feng Huang

**Affiliations:** ^1^Department of Rheumatology, Chinese PLA General Hospital, 28 Fuxing Road, Beijing 100853, China; ^2^State Key Laboratory of Kidney Disease, Chinese PLA General Hospital, 28 Fuxing Road, Beijing 100853, China

## Abstract

**Objectives:**

Ankylosing spondylitis (AS) is a chronic disease that decreases mobility, function, and quality of life. This study introduced the “Smart-phone SpondyloArthritis Management System” (SpAMS), an interactive mobile health (mHealth) tool designed for AS/spondyloarthritis (SpA) disease management and used SpAMS data to evaluate clinical characteristics of Chinese patients with AS.

**Methods:**

SpAMS integrates patient's and physician's portals in a smart phone application. The Chinese Ankylosing Spondylitis Prospective Imaging Cohort was launched using SpAMS in April 2016. Patient self-assessments were completed online at baseline and at every subsequent clinic visit. Physician-reported assessments and treatments were recorded by rheumatologists during each visit.

**Results:**

In total, 1201 patients with AS [mean (SD) age, 30.6 (8.7) years; male, 82.6%] were recruited. Mean (SD) disease duration was 8.4 (6.1) years. Past or current symptoms of acute anterior uveitis (AAU), psoriasis, and inflammatory bowel disease (IBD) were observed in 21.0%, 3.7%, and 9.4% of patients, respectively. AAU and IBD occurred significantly more in patients with symptom duration > 10 years. The most commonly used medications at baseline were nonsteroidal anti-inflammatory drugs (98.2%). Patients using tumour necrosis factor inhibitors accounted for 20.8%, and 66.4% of patients used conventional synthetic disease-modifying antirheumatic drugs. At baseline, 57.2% of patients had inactive disease (ID)/low disease activity (LDA); this rate significantly improved to 79.2% after a mean follow-up of 13.3 (5.9) months. Compared with relapsed patients, new achievers of ID/LDA underwent more online patient assessments (*P *< .001). Problems solved in SpAMS caused 29.1% of clinic visits to a tertiary hospital unnecessary. SpAMS saved an average of 5.3 hours and 327.4 RMB per person on traffic expenses; these expenses equalled 16% of the Chinese monthly disposable personal income.

**Conclusions:**

SpAMS is a time- and cost-saving disease management tool that can help patients with AS perform self-management and provide valuable data to clinicians.

## 1. Introduction

Ankylosing spondylitis (AS) is a chronic inflammatory disease that primarily affects the axial skeleton and is characterised by inflammatory back pain, bony fusion, and new bone formation in the spine, leading to decreased mobility, function, and quality of life [1, 2]. Inflammation in AS may also affect the peripheral joints, entheses, eyes, gastrointestinal tract, heart, and lungs. AS occurs predominantly in young adult males, and disease incidence associates strongly with human leucocyte antigen B27 (HLA-B27).

The prevalence of AS varies widely throughout the world [3]. The total AS prevalence among Chinese residents is 0.3% ~ 0.5% [4, 5], indicating that there are more than 4 million people with AS in China. There is a global challenge in meeting patients' needs and discrepancies in access to AS care [6, 7], highlighting the urgent need to implement cost-effective disease management programs and deliver equal access to care. In addition, China is one of the world's most populous countries, covering an area of 9.6 million square kilometres. Due to a lack of a well-established primary care and specialty referral system and unequal distribution of healthcare resources, most patients with rheumatic diseases are rushed to the metropolis to seek medical care. Owing to the large population and broad geographical distribution of AS patients, the problem of disease management of AS is much more prominent in China. Mobile heath (mHealth) is emerging as a useful and promising tool to improve the convenience of communication between patients and physicians and extend medical care to broader populations in China. In this preliminary analysis, we aimed to describe the design of SpAMS and the clinical characteristics of our cohort, as well as compare our data with those of other global cohorts [8–11].

## 2. Methods

### 2.1. Tool Design

**Figure 1:**
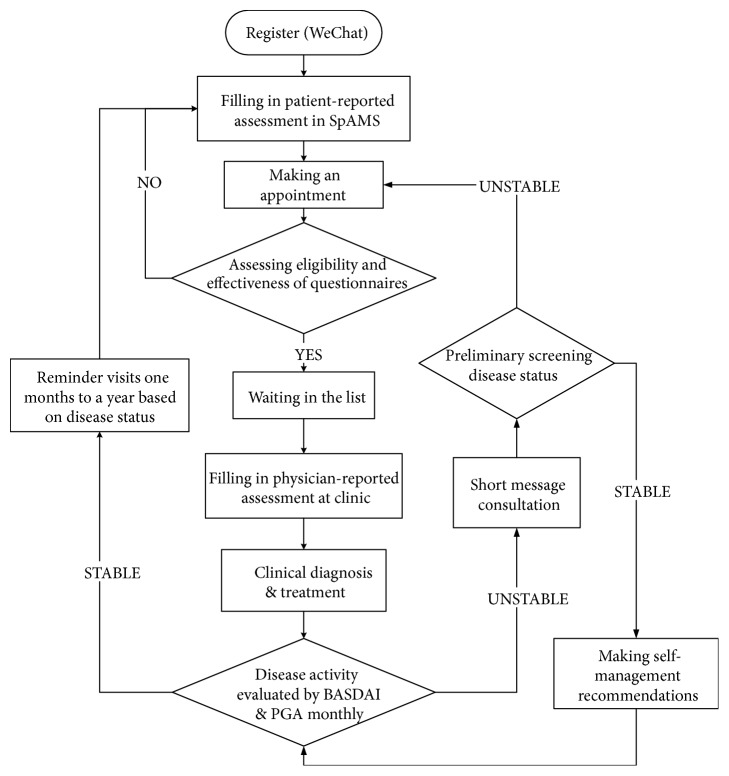
Flow chart of the workflow built into the Smart-phone Spondyloarthritis Management System (SpAMS).

The Smart-phone SpondyloArthritis Management System (SpAMS) was created using mHealth technologies to provide patient education, deliver advice on disease management, and improve medication adherence in patients with AS in China. SpAMS was specifically designed to conduct multicentre prospective studies on spondyloarthritis (SpA)/AS in China using real-world clinical workflows (Figure 1). This management system consisted of a patient mobile terminal (patient's portal), a physician workstation (physician's portal), and a network communication system. The patient's portal was used for performing the following tasks: self-assessment prior to clinic visits, receipt of short message communications, monitoring of symptoms during visit intervals, uploading/downloading of medical records, and receipt of clinic appointment reminders. The physician's portal allows for professional assessments of a patient's disease activities, appointment scheduling, and providing patient-specific recommendations and counselling.

SpAMS links to WeChat, the world's largest multipurpose social media mobile application software (developed by Tencent, first released in 2011). The WeChat Official Account Admin Platform was a service launched for government, hospitals, and private companies as a means of communication with clients. WeChat was used by physicians to maintain communication with patients, record long-term follow-up information, and receive patients' feedback in an economically viable and sustainable fashion. Moreover, using WeChat, SpAMS was developed into a self-sustaining model allowing for efficient physician-patient interactions (Figure 1).

### 2.2. Study Population

The Chinese Ankylosing Spondylitis Prospective Imaging Cohort (CASPIC) study is a nationwide, ongoing, prospective, longitudinal, and state-funded cohort study launched in conjunction with SpAMS. Since databases on AS in China are limited, we enrolled patients without imposing limits on age and disease duration, so as to observe the whole picture and to comprehensively evaluate the characteristics and outcomes of patients with AS in China. All questionnaires used for physician-reported assessments were collected at the Chinese People's Liberation Army (PLA) General Hospital, a tertiary hospital in Beijing, using SpAMS. The 1984 modified New York criteria were used as AS diagnostic criteria [12].

Eligible individuals were patients with AS who had a smart phone with network access. Patients were recruited consecutively from outpatient rheumatology clinics, irrespective of the presence of concomitant acute anterior uveitis (AAU), psoriasis, or inflammatory bowel disease (IBD). Patients who refused to join the online survey or had invalid questionnaires with missing data were excluded.

### 2.3. Collected Parameters

Unified online protocol-directed methods were made. Characteristics of online registration included age, sex, height, weight, smoking status, comorbidities, past medical history, date at onset of back pain, date of diagnosis, presence of AS features, and family history. Follow-up assessments for AS were categorised as patient-reported assessment or physician-reported assessment. The former was collected online prior to the clinic visit. The latter was collected and uploaded at the clinic by the managing rheumatologist. Subsequent visits were scheduled according to the patient's needs (1 month to 12 months) as evaluated at each visit. X-rays (every 2 years), computed tomography (CT) scans, and magnetic resonance images (MRI) of the sacroiliac joint and of the spine were collected for digitisation.

The treatment strategies were determined according to each patient's disease activity states. Nonsteroidal anti-inflammatory drugs (NSAIDs), tumour necrosis factor inhibitors (TNFi), conventional synthetic disease-modifying antirheumatic drugs (csDMARDs), and traditional Chinese medicines were recorded at each visit.

### 2.4. Questionnaires and Disease Evaluation

Standardised questionnaires used for patient-reported assessments included questions on the following:

- The patient's global assessment of disease activity on a numerical rating scale (NRS) of 0-10
- General pain on an NRS of 0-10
- Nocturnal pain levels on an NRS of 0-10
- Bath Ankylosing Spondylitis Disease Activity Index (BASDAI) [13]
- Bath Ankylosing Spondylitis Functional Index (BASFI) [13]
- Work Productivity and Activity Impairment [14]
- Assessment of Spondyloarthritis International Society Health Index (ASAS HI) [15]

Standardised questionnaires used for physician-reported assessments included questions on the following:

- The location of back pain, peripheral arthritis (28 joints), and enthesitis according to the Maastricht Ankylosing Spondylitis Enthesitis Score [16]
- Presence of AAU, psoriasis, and a colonoscopy and pathology-confirmed diagnosis of IBD
- Bath Ankylosing Spondylitis Metrology Index (BASMI) [17] and the chest expansion parameter (the circumferential difference between full inspiration and expiration at the 4th intercostal space)
- Inflammatory markers (erythrocyte sedimentation rate, ESR; C-reactive protein, CRP)
- HLA-B27 status
- Ongoing treatment for AS

Additionally, the Ankylosing Spondylitis Disease Activity Score (ASDAS) was calculated using a formula defined for assessing disease activity in patients with AS [18, 19]. The ASAS-European League against Rheumatism (EULAR) management recommendation for SpA (2016 update) suggested that the preferred measure to define active disease should be the ASDAS, and that active disease can be defined by an ASDAS of at least 2.1 [20]. The disease activity states were defined according to the ASDAS 2018 update [21], which separated inactive disease (ID) from low disease activity (LDA) by 1.3, LDA from high disease activity (HDA) by 2.1, and HDA from very high disease activity (VHDA) by 3.5. According to the baseline ASDAS, patients were divided into two groups: achievers of ID/LDA (ASDAS of less than 2.1) and patients with active disease (ASDAS of at least 2.1). According to the final ASDAS, achievers of ID/LDA were divided into maintainers of ID/LDA and patients with relapse whereas patients with active disease were divided into maintainers of active disease and new achievers of ID/LDA.

Abnormal CRP was defined as CRP > 6 mg/L. When the CRP level was below the limit of detection, a constant value of 2 mg/L was used to calculate the ASDAS [22]. Body Mass Index (BMI) was calculated as the ratio of weight (kg) to height (m) squared.

### 2.5. Quality Control

To enhance the accuracy and reliability of the study, a surveillance approach was implemented.

Quality control for patient-reported assessments was as follows: to ensure that the patient accurately understood the questionnaire information, patients were given verbal instructions, online guidance in SpAMS, and a previsit education session by a qualified nurse. During visits, the physician checked the patient's self-reported questionnaire for any gross errors or irrational data. Erroneous questionnaires were performed again.

Quality control for physician-reported assessments was as follows: a standardised online file system ensured that all investigators used the same protocol-directed methods for data collection.

### 2.6. Ethics Committee Approval

The study protocol was approved by the Ethical Committee of the Chinese PLA General Hospital (S2016-049-02). Informed consent for participation in the study was collected from all patients before study entry.

### 2.7. Statistical Analysis

Considering the influence of disease duration on important outcomes such as BASMI, we divided the enrolled patients into groups based on disease duration at baseline. Data are reported as numbers (%) and means (SD). Statistical analyses were performed using Pearson's chi-square or Fisher's exact test for categorical variables and one-way analysis of variance or Kruskal-Wallis test for quantitative variables. A 2-tailed* P* value < 0.05 was considered as statistically significant. All analyses were performed using Empower (R) (www.empowerstats.com, X&Y Solutions, Inc., Boston, MA) and R (http://www.R-project.org).

## 3. Results

### 3.1. Baseline Clinical Characteristics

From April 2016 to April 2018, a total of 1294 consecutive patients with confirmed AS were registered in the SpAMS database. Ninety-three (7.1%) patients who refused to participate in the online survey (n = 43) or had invalid questionnaires (n = 50) were excluded. In the study cohort, 1201 AS patients from all over the country were enrolled. A total of 4659 patient-reported assessments (mean 3.9, from 1 to 21), including BASDAI and BASFI, and 3304 physician-reported assessments (mean 2.8, from 1 to 11) were collected.

Data from 1201 patients with AS [mean age: 30.6 (8.7) years, male 82.6%, HLA-B27 positive rate 88.9%] were included in the baseline analysis. Baseline clinical characteristics are summarised in Table 1. The mean disease duration was 8.4 (6.1) years. Current or previous history of IBD, AAU, and psoriasis was documented in 9.4%, 21.0%, and 3.7% of the patients, respectively.

Patients with disease durations of ≤ 5 years (384 patients), 5–10 years (420 patients), and > 10 years (397 patients) were compared. Those who smoked and those with pain located in the cervical spine regions were associated with longer disease durations. Frequency of a past history or current symptoms of hip pain was statistically different only in the ≤ 5 years group versus the 5~10-year group, and the ≤ 5 years group versus the > 10 years group. Knee pain was the most prevalent symptom of peripheral arthritis and was 40.5%. It was not associated with disease duration. IBD and AAU were found significantly more frequent in patients with disease duration of > 10 years. The frequency of AAU was statistically different among all the groups. No significant differences were detected in gender, HLA-B27 positivity rate, family history, a current or previous history of psoriasis, current arthritis, and enthesitis in different disease duration groups.

Based on ASDAS, 22.8% patients had inactive disease, and 31.3%, 35.9%, and 9.9% patients had low, high, and very high disease activity at baseline, respectively. Laboratory and disease activity scores are summarised in Table 1. The BASFI, ASAS HI, BASMI, and ESR were significantly higher in the > 10 years group when compared with the ≤ 5 years group and 5~10 years group. A trend toward a significantly higher PGA, PhGA, ASDAS, and BASDAI was observed in the > 10 years group than in the ≤ 5 years group. The CRP levels were similar among the three study groups. However, the frequency of patients with elevated CRP was significantly higher in patients with longer disease durations.

Details of the baseline treatment regimen with various drug combinations and monotherapies are presented in Table 2. Respectively, 98.2% and 66.4% of patients had received NSAIDs and csDMARDs, including sulfasalazine (25.2%), leflunomide (13.5%), methotrexate (3.3%), and thalidomide (23.7%). TNFi, including recombinant human TNF-*α* receptor II:IgG Fc fusion protein and adalimumab, were used in 20.8% of the patients. Sulfasalazine was less used in patients with a disease duration of > 10 years (17.4%,* P* = .001). Patients with long disease duration were more likely to receive thalidomide (Table 2).

NSAIDs as monotherapy were prescribed for 22.5% of patients; TNFi monotherapy was used in 0.6% of the patients. The most common drug combination was NSAIDs plus csDMARDs (56.3%), followed by NSAIDs plus TNFi (10.3%). A combination regimen with three drugs was given to 9.5% of patients.

### 3.2. Changes in Disease Activity

Further analyses were performed for patients with at least two records of ASDAS (at baseline and in the last follow-up). A total of 777 patients with AS were included (Table 3). The rate of patients with ID/LDA (an ASDAS score lower than 2.1) was 57.2% (445/777) at baseline and increased significantly to 79.2% (615/777) with a mean (SD) follow-up of 13.3 (5.9) months. Among patients with ID/LDA at baseline, 92.1% (410/445) maintained the ID/LDA disease activity state. Compared with patients who relapsed, those that maintained the ID/LDA had more patient assessments [5.0 (2.7) versus 3.3 (1.8),* P *< .001] at baseline. In total, 61.7% (205/332) of patients with active disease at baseline achieved ID/LDA. The new achievers of ID/LDA completed more frequent online patient assessments [5.6 (3.1) versus 4.5 (2.5),* P *< .001] compared to patients who maintained active disease. Compared with patients who experienced relapse, new achievers of ID/LDA underwent more frequent patient assessments [5.6 (3.1) versus 3.3 (1.8),* P* < .001], and a higher proportion of these patients were prescribed TNFi during the follow-up period (45.6% versus 27.3%,* P* = .049). However, even in those with persistent active disease, the PGA [4.3 (2.2) versus 3.4 (1.9),* P *< .001], BASDAI [3.4 (1.8) versus 2.6 (1.4),* P *< .001], and BASFI [2.6 (1.8) versus 2.0 (1.6),* P *= .006] improved significantly.

### 3.3. Economic Benefits of SpAMS

From the 1201 patients enrolled in CASPIC, 4659 patient self-assessments were completed, including 3304 previsit assessments and 1355 assessments during the follow-up interval for changes in disease condition. After completing the online self-assessment, patients could receive an online consultation, wherein physicians would advise on the necessity of a follow-up appointment to the Chinese PLA General Hospital based on a patient's past medical records. For patients with problems that could be solved online using SpAMS, 29.1% (1355/4659) of clinic visits to a tertiary hospital were unnecessary. Of the 1201 patients, 86.3% (1037/1201) were not living in the Beijing metropolitan area. As the time and cost of railway travel were relatively stable, we calculated the time and cost of each patient's journey to Beijing based on the location of each patient (based on the China High-speed Railway, 200~350 km/hour). For the 1037 patients who were not living in Beijing, at least 5512 hours (an average of 5.3 hours for each person) and 339,552 RMB (an average of 327.4 RMB for each person; USD: RMB =1:6.418) for traffic time and expenses were saved by using SpAMS. The traffic expenses saved by SpAMS equalled 16% of the Chinese monthly disposable personal income according to the 2016 data from the National Bureau of Statistics of China.

### 3.4. Comparison with Other Cohorts

The main characteristics and disease manifestations of AS were compared among Chinese patients with AS in the current study and other cohorts, including the German spondyloarthritis inception cohort (GESPIC) [8], the Devenir des spondyloarthropaties indifférenciées récentes (DESIR) [9], the Swiss clinical quality management cohort (SCQM) [10], and the outcome in ankylosing spondylitis international study (OASIS) [11] (Table 4). In all studies, males were more frequently affected than females. Our data showed a mean age at onset of 22.2 (7.7) years and 30.6 (8.7) years at study entry, which was lower than that reported for cohorts in Western countries. The percentages of HLA-B27 positivity in our cohort were comparable to other cohorts. The extra-articular manifestations and treatment regimes varied by geographic location.

## 4. Discussion

### 4.1. Principal Results

Based on several real-world studies and web-based registries encompassing large and heterogeneous groups of patients, many centres have conducted clinical research with prospective cohorts that include long-term follow-up [23–25]. Real-world studies capitalise on the exponential growth in access to data from multiple sources, such as mHealth applications, electronic health records, claims and billing data, and information from social media [23, 26]. Among these sources, mHealth is a particularly useful source for observational studies in real-world settings. In contrast to real-world studies, randomised controlled trials (RCTs) are conducted in highly selective patient groups in ideal conditions, which may limit the translation of results into the complex situations that occur in real clinical practice. Real-world studies using mHealth technology have the potential to supplement the results of RCTs and have several unique advantages. Firstly, the use of mHealth makes it possible to comprehensively evaluate patients' disease activity status in a broader population [27]. Secondly, mHealth can also support patients' self-tracking and disease management through mobile devices and health applications. These features of mHealth tools have made real-world healthcare studies economically and technically feasible. In the present study, a real-world setting and mHealth technology were used to establish a prospective and population-based database in Chinese patients with AS.

During the two years of the registry, 1201 patients were enrolled in CASPIC. To ensure consistency of the data, all questionnaires used for physician-reported assessments were collected at the Chinese PLA General Hospital. The CASPIC is the first mHealth registry of AS in China, aimed primarily at forming a prospective and comprehensive observational cohort to study the major clinical characteristics and outcomes of AS. These findings have the potential to facilitate the development of research in epidemiology, medicoeconomy, disease management, and prognosis. Various cohort studies on AS and/or Spondyloarthritis (SpA) have been performed worldwide and have provided valuable data to help improve the prognosis and quality of life of AS patients. Published percentages of male patients among the AS patient population range from 65% to 80% and vary by geographic location [28]. The percentages of HLA-B27-positive patients with AS have been reported to be 90-95% [29]. The prevalence of peripheral arthritis in Chinese patients with AS has been reported to be 50% [30], which is similar to that observed in our study (69.0% with hip pain and 40.5% with knee pain). Extra-articular manifestations of AS have been reported worldwide with an extremely varied racial and ethnic prevalence. AAU is the most frequent extra-articular feature in AS [28], and its prevalence varies by geographic location. A systematic literature review based on patients with AS revealed a mean prevalence of AAU of 3.2%, which increased with disease duration [31]. In our study, 20.1% patients presented with one or more flare-ups of AAU and the prevalence was significantly related to the duration of disease.

In our study, the treatment of patients was determined by the discretion of the rheumatologist. Antirheumatic treatments were categorised as NSAIDs, csDMARDs, TNFis, and traditional Chinese medicines. TNFi therapy is the most effective therapy for AS and SpA and is the recommended second-line treatment for individuals with persistent high disease activity despite treatment with at least two NSAIDs and for those with intolerance or contraindications to NSAIDs [20]. Noticeably, as most medical insurances in China do not fully cover the cost of TNFi, most patients may not be able to afford the high cost of TNFi therapies for a prolonged time. This factor is thus a major impediment to the widespread use of TNFi therapy in China. Only 20.8% of patients in our cohort were TNFi-users at baseline, and 29.9% used TNFi at some point of time during the observation period. Sulfasalazine is the most extensively used csDMARDs in the treatment of AS and the most used csDMARDs (25.2%) in this cohort. The 2016 update of the ASAS/EULAR management recommendations for axial SpA suggests that sulfasalazine may be considered in patients with peripheral arthritis [20]. Thalidomide has a long history in the treatment of AS in China (from 1990s) and has been shown to have good efficacy [32, 33] and long-term safety [34] for the treatment of AS. Due to the reproductive toxicity of thalidomide, we strictly followed the system for thalidomide education and prescribing safety (STEPS) programme [35]. This also explains why the rate of thalidomide is much lower in patient with shorter disease duration as shown in Table 2 (P < .001).

With the increasing global popularity of smart-phone technology, there is a great opportunity for patients with AS to use mHealth applications for disease management and communication with physicians [36]. In the field of rheumatoid arthritis (RA), a Japanese study showed that the disease activity of RA can be accurately assessed by a smart-phone application [37]. More importantly, mHealth disease management tools are increasingly recognised as critical to improving health outcomes and may lead to the formation of effective therapeutic relationships for patients with rheumatic disease [7, 37, 38]. One study showed that patients were more active in self-management participation with the mHealth tool [38]. Online patient self-assessment tools can help with patient-centred disease management by helping physicians and patients to monitor disease status at home. The SpAMS presented in the current study is an online patient self-management tool which allows patients to perform self-assessments, receive self-management recommendations, and make clinic appointments according to their disease activity status. From April 2016 to April 2018, 5600 people were registered in SpAMS. Among them, 1449 patients with inflammatory low back pain made 4025 clinic visits in two years, with an average of 2.8 visits per patient. For patients enrolled in CASPIC, the rate of patients with ID/LDA was increased significantly after disease management with SpAMS. Even in patients who failed to achieve ID/LDA, the PGA, BASDAI, and BASFI scores were improved significantly. Notably, patients who achieved or maintained ID/LDA completed more online self-assessments. After completing the self-assessment using SpAMS, patients were allowed to receive an online consultation. Their physicians would advise on whether it was necessary to make a follow-up clinic appointment, based on their assessment. For patients with problems that could be solved at home, more than a quarter of clinic visits were unnecessary and the time that would have been used for these visits was thus saved by SpAMS. Therefore, SpAMS could serve as a cost- and time-saving specialty care online platform for patients with AS.

### 4.2. Comparison with Prior Work

To date, SpAMS has successfully been a user friendly, patient-engaging mHealth tool that sustainably generates data suitable for real-world clinical research. More importantly, SpAMS may be able to differentiate between patients in regular clinics and those who are selectively interested in self-assessments or self-management strategies. This distinction can improve the accuracy of data used for scientific research and is important for mHealth tools which serve as data sources for research purposes.

SpAMS had a high patient adherence rate. It was previously reported that the adherence rate varied from 30% to 75% [39]. In CASPIC, 860 (71.6%) patients visited the clinic at least 2 times in two years. In total, 447 (37.2%) patients were enrolled within one year prior to the end date of data inclusion for this study. According to the follow-up interval (1 month to 12 months) that was set, some of those patients may have not yet had a follow-up visit, which may contribute to a drop in the adherence rate. With the exclusion of these patients, the adherence rate increased to 87.7% (754/860). Based on feedback provided during visits, patients were in favour of this system, which continuously monitored and recorded their health conditions, including disease activity, medication changes, and other AS-associated conditions (e.g., AAU/IBD) and made these data available for review at any time. Additionally, we devised other ways to improve adherence. First, once patients completed their medical visits, a personalised summary was generated and sent to the patient. The summary included the patient's health status, treatments, and the recommended date for their next medical visit. Second, a personalised reminder system was developed to remind patients of the appointment schedule two weeks before the recommended visit date. Third, SpAMS was helpful in providing comprehensive information on self-management. This is important because physicians in China have an extremely heavy workload in the outpatient setting. For example, at the Rheumatology Department of Chinese PLA General Hospital, each physician needs to see an average of 40 patients every half work day, which corresponds to less than 10 minutes spent per patient with their physician. Such a short time allotted per patient may be not sufficient to communicate and deliver comprehensive counselling and disease education to patients. In April 2018 alone, 40151 social media followers of this application perused the self-management strategies up to 68296 times, at an average of 1.7 times per person per month. Many study participants provided valuable feedback and commented on the usefulness of SpAMS as a self-management tool. Lastly, SpAMS provided patients with opportunities for connecting with their treating physicians remotely, which is one of the perceived needs of patients with inflammatory arthritis [7]. The self-assessment and self-management aid provided by SpAMS is personalised and patient-tailored, which can help patients and generate a wealth of socioeconomic benefits.

### 4.3. Limitations

The main strengths of our study are the prospective real-world design, including an unselected sample of AS patients in China, the mHealth data collection method. However, user-orientated platforms always present challenges related to data collected by subjective assessments [37]. We implemented many strategies to avoid bias, such as providing online instructions in SpAMS, previsit nurse-administered education sessions, autocorrect functions for data collection, and physician review during visits. Only patients with data that passed all the above-mentioned quality check procedures were enrolled in the CASPIC. However, some potential bias and lack of accuracy in data collection may still be present. Therefore, some of the most important data were collected via the physician's portal (the physician's entry system during visits) during face-to-face assessments.

## 5. Conclusions

In conclusion, this observational cohort study was the first nationwide real-world study in China to characterise patients with AS using mHealth technology. These study results may help improve our knowledge about the characteristics of AS. With disease management using SpAMS, the rate of patients with ID/LDA increased significantly, and the patients who achieved or maintained ID/LDA completed more online self-assessments. SpAMS is a time- and cost-saving disease management tool that can serve as a specialty care online platform, ultimately benefiting both patients and physicians.

## Figures and Tables

**Table 1:** Baseline characteristics of the patients with ankylosing spondylitis (AS) categorised by disease duration.

Characteristics	All patients with AS (n =1201)	Duration≤5 years (n = 384)	Duration>5 years and ≤10 years (n = 420)	Duration > 10 years (n = 397)	*P* value^a^
Age, mean (S.D.), years	30.6 (8.7)	26.8(8.0)	29.3(7.3)	35.7(8.5)	<0.001*∗*
Age at disease onset, mean (S.D.), years	22.2 (7.7)	24.2 (8.1)	22.0 (7.2)	20.5 (7.4)	<0.001*∗*
Disease duration, mean (S.D.), years	8.4 (6.1)	2.6 (1.4)	7.3 (1.4)	15.2 (5.2)	<0.001*∗*
Male gender, %	82.6	79.4	84.0	84.1	0.139
HLA-B27 positive, %	88.9	88.6	89.9	88.3	0.748
Smoker, %	33.0	28.1	33.5	37.2	0.025*∗*
Family history of AS, %	25.3	23.9	24.1	28.1	0.317
Past history or current symptoms of, %					
Cervical spine pain	53.3	35.8	52.9	70.5	<0.001*∗*
Hip pain	69.0	63.1	70.7	72.8	0.010*∗*
Knee pain	40.5	38.8	40.0	42.7	0.525
AAU	21.0	12.9	19.0	31.1	<0.001*∗*
IBD	9.4	6.3	9.4	12.5	0.013*∗*
Psoriasis	3.7	3.4	3.9	3.8	0.935
Physical examination, %					
Peripheral arthritis	13.6	14.0	12.3	14.5	0.706
Enthesitis	23.7	25.3	24.1	21.8	0.608
PGA, mean (S.D.)	3.0(2.1)	2.8 (2.1)	3.0 (2.1)	3.3 (2.2)	0.004*∗*
PhGA, mean (S.D.)	2.2 (1.4)	2.1 (1.3)	2.1 (1.3)	2.4 (1.4)	<0.001*∗*
BASDAI, mean (S.D.)	2.4 (1.7)	2.2 (1.7)	2.3 (1.7)	2.6 (1.8)	0.003*∗*
BASFI, mean (S.D.)	1.6 (1.7)	1.4 (1.6)	1.5 (1.6)	2.0 (1.9)	<0.001*∗*
BASMI, mean (S.D.)	1.7 (2.0)	1.0 (1.7)	1.4 (1.9)	2.5 (2.2)	<0.001*∗*
ASAS HI, mean (S.D.)	5.4 (3.8)	5.2 (3.6)	5.1(3.8)	5.8 (3.9)	0.041*∗*
CRP, mean (S.D.), mg/L	14.0 (24.6)	12.3 (21.8)	13.9 (21.7)	15.7 (29.5)	0.257
Elevated CRP, %	48.2	41.4	46.8	56.1	0.002*∗*
ESR, mean (S.D.), mm/hour	16.5 (17.8)	14.2 (16.0)	16.7 (19.2)	18.3 (17.7)	0.018*∗*
Elevated ESR, %	26.7	21.9	27.9	29.9	0.072
ASDAS, mean (S.D.)	2.2(1.0)	2.0 (1.0)	2.1 (1.0)	2.3 (1.0)	<0.001*∗*

AS: ankylosing spondylitis, PGA: Patient's global assessment, PhGA: Physician's global assessment, BASDAI: Bath Ankylosing Spondylitis Disease Activity Index, BASFI: Bath Ankylosing Spondylitis Functional Index, BASMI: Bath Ankylosing Spondylitis Metrology Index, ASAS HI: The Assessment of Spondyloarthritis international Society Health Index, ESR: erythrocyte sedimentation rate, CRP: C-reactive protein, ASDAS: Ankylosing Spondylitis Disease Activity Score, HLA: human leukocyte antigen, AAU: acute anterior uveitis, and IBD: inflammatory bowel disease. *∗P* < 0.05.

**Table 2:** Treatment regimens with various drug combinations in patient groups based on different disease durations.

Characteristics	All patients with AS (n=958)	Duration≤5 years (n=305)	Duration>5 years and ≤10 years (n=332)	Duration >10 years (n=321)	*P* value^a^
NSAIDs, %	98.2	97.7	99.1	97.8	0.329
TNFi, %	20.8	18.7	22.3	21.2	0.522
csDMARDs, %	66.4	65.6	68.1	65.4	0.723
Sulfasalazine	25.2	32.8	25.6	17.4	0.001*∗*
Leflunomide	13.5	15.1	13.0	12.5	0.595
Methotrexate	3.3	5.2	2.7	2.2	0.075
Thalidomide	23.7	13.8	25.0	31.8	<0.001*∗*
TCM, %	62.4	61.0	63.0	63.2	0.819
Treatment regimen, %					
NSAIDs monotherapy	22.5	26.0	20.3	21.6	
TNFi monotherapy	0.6	1.0	0.3	0.6	
NSAIDs + csDMARDs	56.3	54.6	57.9	56.2	
NSAIDs + TNFi	10.3	7.2	11.5	11.9	
NSAIDs + TNFi + csDMARDs	9.5	10.2	10.0	8.4	

AS: ankylosing spondylitis; NSAIDs: nonsteroidal anti-inflammatory drugs; TNFi: TNF inhibitors; csDMARDs: conventional synthetic disease modifying antirheumatic drugs; TCM: traditional Chinese medicine. *∗P* < 0.05.

**Table 3:** Characteristics of disease activity at baseline and a mean (SD) follow-up of 13.3 (5.9) months.

Characteristics	ID/LDA at baseline (n=445, 57.2%)			Active at baseline (n=332, 42.7%)		
	Maintainer of ID/LDA (n=410, 92.1%)	Patients with relapse (n=35, 7.9%)	*P* value	Maintainer of active disease (n=127, 38.2%)	New achiever of ID/LDA (n=205, 61.7%)	*P* value
Age, mean (S.D.), years	30.2 (9.1)	29.8 (7.6)	0.785	30.8 (8.6)	30.6 (8.3)	0.816
Disease duration, mean (S.D.), years	7.6 (6.2)	7.3 (5.6)	0.787	9.0(6.1)	9.3(6.1)	0.714
Number of self-assessments, mean (S.D.)	5.0 (2.7)	3.3 (1.8)	<0.001*∗*	4.5(2.5)	5.6(3.1)	<0.001*∗*
Male sex, %	79.8	88.6	0.207	89.8	83.9	0.133
ASDAS at baseline, mean (S.D.)	1.4 (0.5)	1.5 (0.4)	0.031*∗*	3.2 (0.8)	3.0 (0.8)	0.134
Smoker, %	24.7	40.0	0.047*∗*	44.0	32.8	0.042*∗*
NSAIDs at baseline, %	98.1	99.8	0.448	97.9	99.4	0.263
TNFi at baseline, %	19.2	13.8	0.479	24.5	29.8	0.359
csDMARDs at baseline, %	64.0	72.4	0.363	66.0	68.5	0.679
TNFi during the follow-up period^a^, %	29.9	27.3	0.755	32.3	45.6	0.016*∗*

^a^TNFi during the follow-up period: used TNFi at any point beginning from the time of enrolment in the registry (the first visit) and during the follow-up visits. ID: inactive disease, LDA: low disease activity, ASDAS: Ankylosing Spondylitis Disease Activity Score; NSAIDs: non-steroidal anti-inflammatory drugs; TNFi: TNF inhibitors; csDMARDs: conventional synthetic disease modifying anti-rheumatic drug; *∗P* < 0.05.

**Table 4:** Comparison of baseline demographics of ankylosing spondylitis (AS) patients in observational cohort studies.

Demographics	GESPIC (2009)[8]	DESIR (2011)[9]	SCQM (2014)[10]	OASIS (2014)[11]	CASPIC (Our study)
Analysis population	AS <10 years (n=236)	AS <10 years (n=181)	AS (n=838)	AS (n=184)	AS (n=1201)
Geographical area	German	France	Swiss	Netherlands, Belgium, and France	China
Male gender	64.0%	58.6%	74.1%	70%	82.6%
Age, years	35.6(10.2)	31.3(9)	NR	43(12)	30.6 (8.7)
Age at disease onset, years	30.4(10.6)	NR	24.2 (19.7-30.5)	NR	22.2 (7.7)
Disease duration	5.2(2.7) years	19(10.1) months	12.7(6.4-22.7) years	11(9) years	8.4 (6.1) years
HLA-B27 positive rate	82.2%	72.4%	82.5%	83%	88.9%
PGA	5.0(2.5)	NR	6.0(3.0-7.0)	3.7(2.3)	3.0 (2.1)
PhGA	4.5(2.0)	NR	4.0(2.0-5.0)	3.7(2.3)	2.2 (1.4)
BASDAI	4.0(2.1)	4.0(2.1)	4.8(3.0-6.4)	3.4(2)	2.4 (1.7)
ASDAS	NR	2.6(1.1)	3.2(2.3-4.0)	2.6(1.0)	2.2 (1.0)
CRP, mg/L	14.8(16.0)	11.4(15.2)	8.0(5.0-18.0)	17.4(23.3)	14.0 (24.6)
ESR, mm/hour	21.7(18.0)	NR	14.0(6.0-28.0)	14.0(15.0)	16.5 (17.8)
BASFI	3.1(2.5)	2.8(2.2)	3.3(1.5-5.5)	NR	1.6 (1.7)
BASMI	2.0(1.8)	2.2(0.9)	2.0(1.0-4.0)	NR	1.7 (2.0)
Manifestations, ever					
AAU	20.9	11.1	24.1	NR	21.0
IBD	2.6	7.2	9.3	NR	9.4
Psoriasis	10.2	14.4	7.4	NR	3.7
Drug treatment					
NSAIDs	64	71.3	82.3	95(during follow-up)	98.2
TNFi	1.7	NR	20.3	22(during follow-up)	20.8
Methotrexate	8	NR	9.7	NR	3.3
Sulfasalazine	17	NR	6.0	NR	25.2
Leflunomide	NR	NR	1.4	NR	13.5

Values are expressed as mean (or median in the SCQM study) or percentage of patients. Age was defined as age at inclusion in cohort. AS: Ankylosing Spondylitis; GESPIC: German Spondyloarthritis Inception Cohort; SCQM: Swiss Clinical Quality Management Cohort; DESIR: the Devenir des spondyloarthropaties indifférenciées récentes; OASIS: the outcome in ankylosing spondylitis international study; CASPIC: Chinese Ankylosing Spondylitis Prospective Imaging Cohort; HLA: human leucocyte antigen; BASDAI: Bath Ankylosing Spondylitis Disease Activity Index; ASDAS: Ankylosing Spondylitis Disease Activity Score; CRP: C-reactive protein; ESR: Erythrocyte sedimentation rate; BASFI: Bath Ankylosing Spondylitis Functional Index; BASMI: Bath Ankylosing Spondylitis Metrology Index; AAU: acute anterior uveitis; IBD: inflammatory bowel disease; NR: not reported; PGA: Patient's global assessment; PhGA: Physician's global assessment; NSAIDs: nonsteroidal anti-inflammatory drugs; TNFi: TNF inhibitors.

## Data Availability

The data used to support the findings of this study are available from the corresponding author upon request.
